# Special issue: nerve compression syndromes *“Brachioradialis, or “High Wartenberg”,* syndrome – compression of the sensory branch of the radial nerve in the proximal forearm

**DOI:** 10.1007/s00264-024-06350-x

**Published:** 2024-10-17

**Authors:** Elisabet Hagert, Camila Azocar, Ulrika Jedeskog, Ashraf Hantouly

**Affiliations:** 1 Aspetar Orthopaedic and Sports Medicine Hospital, Sports City Street, Al Buwairda St, 29222 Doha, Qatar; 2 Department of Health and Medical Sciences, College of Medicine, Qatar University, Doha, Qatar; 3 Department of Clinical Science and Education, Karolinska Institutet, Sodersjukhuset, Stockholm, Sweden; 4 Department of Hand Surgery, INDISA Clinica, Santiago, Chile; 5 Idrottskliniken Rehab, Solna, Sweden

**Keywords:** Mononeuropathies, Radial nerve, Nerve compression syndromes, Peripheral nerves, Wartenberg syndrome

## Abstract

**Purpose:**

Compression of the sensory branch of the radial nerve (SBRN) in the proximal forearm is an uncommon condition, leading to both motor and sensory deficits. The aim of this study is to assess the surgical outcomes of SBRN release at the level of the brachioradialis arcade.

**Methods:**

A retrospective study of prospectively collected data was conducted on patients undergoing brachioradialis release (BRR) from March 2014 to March 2021. The measured outcomes included quick-DASH (Disability of the Arm Shoulder Hand questionnaire), work-DASH, visual analog scale (VAS) scores for pain, and patient satisfaction with surgery, at a minimum six month follow-up.

**Results:**

A total of twenty patients (mean age of 44.1 (range 25–62) were included in this study, of which nine (45%) were males. Eleven patients (55%) underwent isolated BRR, while the other nine patients (45%) underwent concomitant BRR and lacertus release. The three most common presenting symptoms in patients with isolated BRS were radiovolar forearm pain (100%), disturbed sensation in the SBRN territory (85%), and hand/thumb fatigue (75%). Forearm pain and fatigue were found in all patients with combined BRS and lacertus syndrome. The response rate for the functional outcome scores was 65% (13/20). Quick-DASH significantly improved (preoperative 29.6 (range 13.6–57.5) to postoperative 6.9 (range 0–27.27), *p* < 0.0001) as did the work DASH (*p* < 0.0001). Follow-up VAS Pain was 1 and satisfaction with surgery 9.6.

**Conclusion:**

BRS is an uncommon radial nerve compression syndrome in the proximal forearm that differs from the more commonly recognized radial tunnel syndrome. It presents with radio-volar forearm pain, disturbed sensation in the SBRN distribution, and loss of hand/thumb endurance. Minimally invasive BRR immediately restores wrist extension strength, significantly improves DASH scores, and yields positive outcomes at a minimum six-month follow-up.

**Supplementary Information:**

The online version contains supplementary material available at 10.1007/s00264-024-06350-x.

## Introduction

Nerve compression syndromes remain the most commonly encountered pathologies in hand surgery practice. Of these, the carpal tunnel and cubital tunnel syndromes are frequent, while compression of the radial nerve remains more elusive, regardless of the level of nerve compression.

Compression of the sensory branch of the radial nerve (SBRN), was first described by Wartenberg in 1932, who at the time coined this compression as “*cheiralgia paresthetica”*, denoting the sensory disturbance associated with this nerve compression. Since then, the compression of the SBRN is more commonly known as Wartenberg’s syndrome and implicates a compression of the SBRN in the distal forearm [1].

**Fig. 1:**
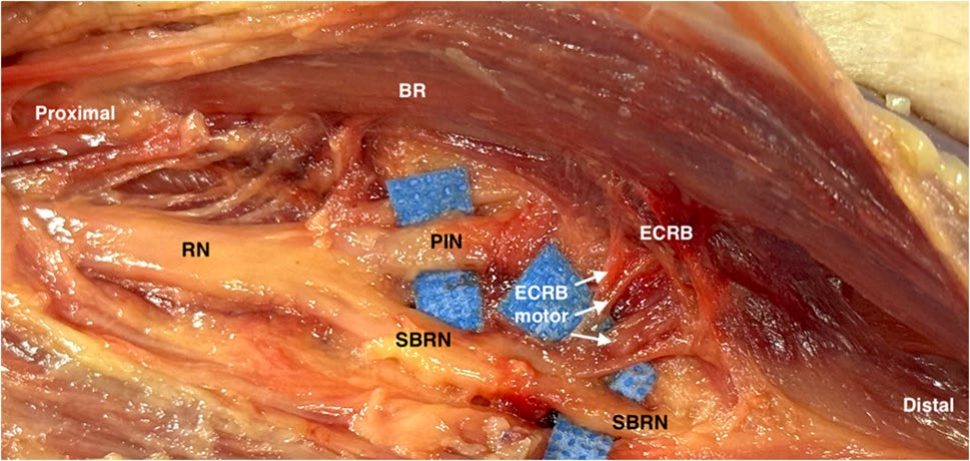
Radial nerve anatomy in the proximal forearm. RN = Radial Nerve; BR = Brachiorradialis; PIN = Posterior Interosseous Nerve; SBRN = Sensory Branch of the Radial Nerve; ECRB = Extensor Carpi Radialis Brevis

The general innervation and branching pattern of the radial nerve (RN) from the level of the elbow to the forearm has been debated, with discussions regarding the motor innervation to the forearm muscles from the RN, the posterior interosseous nerve (PIN) and the SBRN. Although the SBRN is traditionally regarded as a pure sensory nerve, providing sensation to the distal radial forearm, dorsal thumb, index, and middle fingers, recent anatomical studies have provided support that the motor innervation to the extensor carpi radialis brevis (ECRB) is frequently seen to arise from the SBRN [2] (Fig. 1).

This study aims to evaluate the clinical presentation of SBRN compression in the proximal forearm and assess the outcomes following surgical release at a minimum 6-month follow-up.

## Material and methods

This study involving human participants was in accordance with the ethical standards of the 1964 Helsinki Declaration and its later amendments. Ethics committee approval and due consent were also obtained. A retrospective study of prospectively collected data was conducted on the patient registry from March 2014 to March 2021. Patients were included for final evaluation if they had undergone surgical decompression of the sensory branch of the radial nerve at the level of the BR (brachioradialis release, BRR), with or without simultaneous concomitant lacertus release. Exclusion criteria included only conservative management and other prior radial nerve injury or compression.

Medical records were reviewed to collect data on sex, age, occupation, hand dominance. Other clinical variables that were reviewed included subjective symptoms, surgical intervention, and intraoperative return of strength. Functional outcomes included pre- and postoperative quick-DASH (Disability of the Arm Shoulder Hand questionnaire) with work sub-scores, and intraoperative return of strength.

### Brachioradialis syndrome diagnosis (video 1)

The diagnosis of BRS was based on thorough patient history and clinical examination, including a clinical triad of combining upper extremity manual muscle testing (MMT) revealing weakness in the extensor carpi radialis brevis (ECRB) (and in no other radial nerve innervated muscles), provocative sensory testing (scratch collapse test, SCT; loss of sensory perception in the SBRN distribution) and the presence of pain and/or positive Tinel's test at the level of nerve compression [3]. For BRS, pain is present at the level of the SBRN approximately 2 cm distal of the SBRN/PIN bifurcation and about 3 cm distal of the radiovolar elbow crease. Concomitant LS was defined by the Hagert clinical triad of flexor carpi radialis (FCR), flexor pollicis longus (FPL), flexor digitorum profundus index (FDP II) weakness; positive SCT at the lacertus and pain on the same level [4]. Ultrasound was routinely done in the clinic to rule out ganglion cysts or other sources of compression. No electromyographic studies were implemented in diagnosing BRS.

### Surgical technique

Decompression of the SBRN at the level of the BR arcade (brachioradialis release, BRR) was performed under wide-awake, local anaesthesia, no tourniquet (WALANT). The technique followed the principles documented for other proximal forearm WALANT releases (lacertus release, cubital tunnel release) [5] and takes advantage of the possibility to perform intraoperative testing of the return of muscle power.

**Fig. 2:**
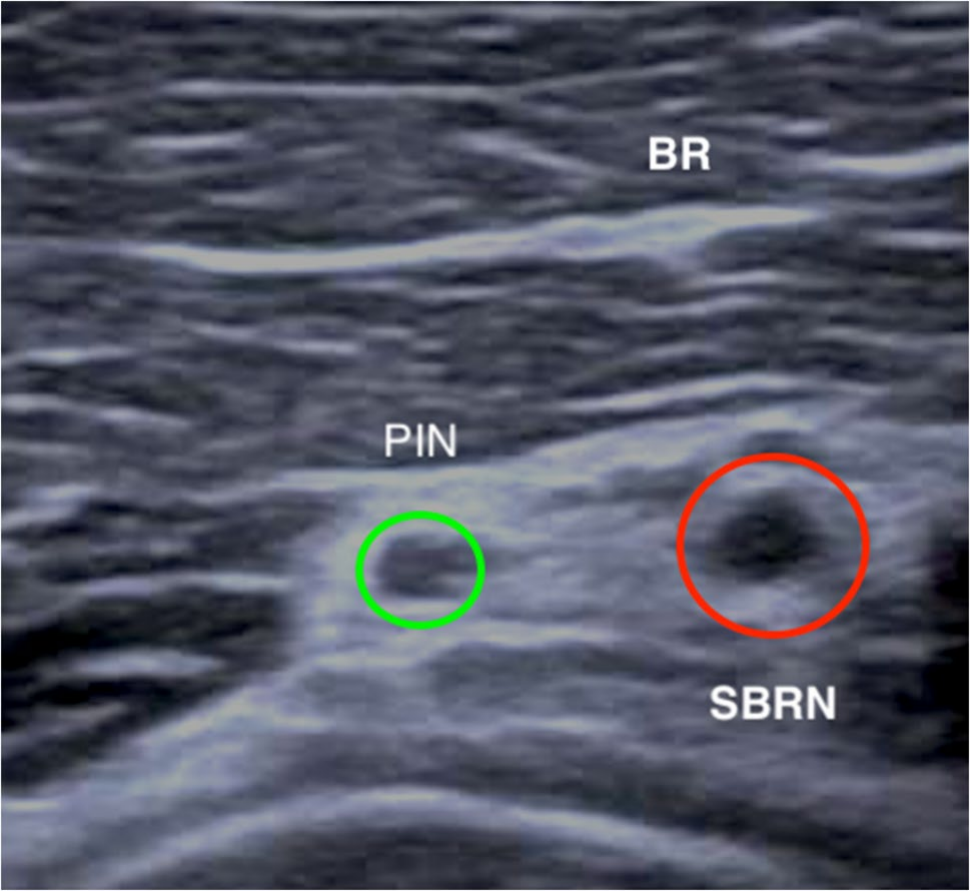
Ultrasound image of the compression site, showing an increased cross-sectional area and hypoecogenicity in the SBRN, as signs of a focal nerve compression. BR = Brachioradialis; PIN = Posterior interosseous nerve; SBRN = Sensory branch of the radial nerve

20–40 cc of lidocaine 1% with epinephrine (0.01 mg) were used, the higher volume in cases of concomitant lacertus release. In cases of isolated BRR, the surgery was performed through a 2–3 cm transverse skin incision in the radiovolar proximal forearm, approximately 2 cm distal of the radiovolar elbow crease. Following blunt dissection to the ulnar border of the brachioradialis, the radial nerve is identified and traced distally to the bifurcation of the posterior interosseous nerve (PIN) and SBRN. Continuing to follow the SBRN, the overlying BR tendon is released as this is found to be the compression point of the SBRN, proximal of the motor branches to the ECRB (Fig. 2). Following adequate release of the SBRN, intraoperative return of wrist extension power is noted. The wound is closed with intracutaneous monocryl 4–0 sutures and a small dressing applied. Immediate range of motion exercises are allowed, avoiding load more than 1–2 kg for the first two weeks after surgery. Return to full load is typically allowed one month postop.

### Outcome measures

Outcome measures were prospectively collected via mail at six months postoperatively by an independent investigator outside the surgical team. A second follow-up letter was sent nine months postoperatively if no response was received after the first. The primary outcomes were the pre-and postoperative quick-DASH with work sub-scores. The DASH questionnaire is a validated tool for standard assessment of the impact on the function of various musculoskeletal diseases and injuries in the upper extremity; scores range from 0 to 100, with higher ranking indicating worse symptoms. Secondary outcomes included intraoperative return of strength through MMT and patient feedback.

### Statistical analyses

Patient demographics were collected. Pre- and postoperative scores were compared using paired two-tailed Student's t-test. *P* values of < 0.05 were considered statistically significant. Numbers (iWork, Apple Inc, v.14.1) was used to perform the statistical analysis.

## Results

A total of twenty patients with SBRN release at the level of the brachioradialis arcade (BRR) were included over seven years. The cohort consisted of eleven females and nine males with a mean age of 44.1 (range 25–62). Of these, eleven cases (55%) were isolated BRR, and nine cases (45%) were concomitant BRR and lacertus release. Three patients who underwent isolated BRR had undergone prior lacertus release with a resolution of median nerve symptoms but with onset of BRS approximately two years later. Patients' demographics are summarized in a table (Table 1).

**Table 1:** Patients demographics

Demographics		
Patient Variables	No	
Age (year), mean (range)	44 (25 − 62)	
Sex, No (%)	Female = 11 (55%)	Male = 9 (45%)
Arm Affected, No (%)	Dominant = 14 (70%)	Non-dominant = 6 (30%)
Office Worker No (%)	18 (90%)	
Concomitant lacertus syndrome	9 (45%)	

### Presenting symptoms

The three most common presenting symptoms in patients with isolated BRS were radiovolar forearm pain (100%), disturbed sensation in the SBRN territory (85%), and hand/thumb fatigue (75%). In patients with combined BRS and LS, the predominant symptoms were of general forearm pain (100%) and loss of hand strength/endurance (100%).

### Surgical outcomes

Pre- and postoperative quick-DASH with work sub-scores were obtained from 13 patients, as were postoperative visual analogue scale (VAS, Likert Scale 0–10) scores on pain and satisfaction with surgical outcomes. The average preoperative quick DASH was 29.6 (range 13.6–57.5); and work DASH 41.1 (range 0–93.75). The average postoperative quick DASH was 6.9 (range 0–27.27), which is a statistically significant reduction *(p* < 0.0001). Similarly, the work DASH subscore was significantly reduced *(p* < 0.001) to 13.6 (0–31.23). The average postoperative VAS Pain score was 1 and satisfaction with surgery 9.6 (0- not happy, 10- very happy).

All cases were done using WALANT anesthesia which allows immediate evaluation of return of power in the ECRB, and in the instance of concomitant lacertus release, of the FCR, FPL, and FDP II. Intraoperative return of strength, as measured using MMT and patient feedback, was verified in all patients.

## Discussion

This study evaluated 20 patients who underwent sensory branch radial nerve (SBRN) release, with 55% receiving isolated brachioradialis release (BRR) and 45% undergoing concomitant BRR and lacertus release. The most common presenting symptoms in patients with isolated brachioradialis syndrome (BRS) were radiovolar forearm pain, sensory disturbances in the SBRN distribution, and hand/thumb fatigue. All patients with combined BRS and lacertus syndrome experienced forearm pain and fatigue. Postoperatively, significant improvements were observed in Quick-DASH and Work-DASH scores, with a mean VAS pain score of 1 and a high satisfaction rate at a mínimum six month follow-up.

As this type of nerve compression involves the SBRN, it could thus be named a “high Wartenberg” syndrome; a “low Wartenberg” being the SBRN compression in the distal forearm (Table 2). While eponyms can be beneficial in remembering a diagnosis or associating syndromes, they suffer from a lack of detail and precision [6]. The compression of the SBRN in the proximal forearm is therefore recommended to be called “*brachioradialis syndrome”* as this is the primary cause of compression.

**Table 2:** Clinical findings in low vs high Wartenberg syndrome

	Pain	Sensory exam—Numbness/ Dysesthesia	Motor Exam -Weakness
”Low” Wartenberg Syndrome	Radial distal forearm Radial wrist	Dorsoradial hand-thumb to middle finger	No weakness—normal muscle func:on
”High” Wartenberg Syndrome	Proximal radial forearm Radia:ng into distal forearm/ radial hand/thumb	Radiovolar forearm Dorsoradial hand	ECRB

Compression of the SBRN in the proximal forearm has previously been reported in in association with radiocapitellar ganglion cysts [7] as well as aberrant branches of the radial artery causing compression [8]. These reports are based on smaller case series or simple case reports, where the patient suffers from pain and sensory symptoms but no reported loss of motor function.

The associated motor loss seen in the brachioradialis syndrome is that of loss of wrist extension power due to weakness in the ECRB. The motor innervation of the ECRB was first described by Sunderland in 1947, arising from the radial nerve, the “superficial radial” (SBRN), the PIN, or a combination of these three [9]. Later studies have looked at the details regarding the innervation pattern and branches to the extensor muscles of the forearm, concluding that the ECRB is innervated solely from the SBRN in approximately 25% of cases, where the motor branches are found distal of the PIN-SBRN bifurcation point [2, 10, 11].

Surgical decompression for “Low Wartenberg” syndrome with a complete distal brachioradialis tenotomy has been described with good clinical outcomes [12]. This series shows similar results when treating BS with a BRR at the proximal forearm where the compression site is located. As proximal SBRN compression can cause ECRB motor loss, and the surgeries were performed under WALANT anesthesia, we could check the immediate strength recovery of wrist extension during surgery in all cases, confirming intraoperative complete decompression.

**Fig. 3:**
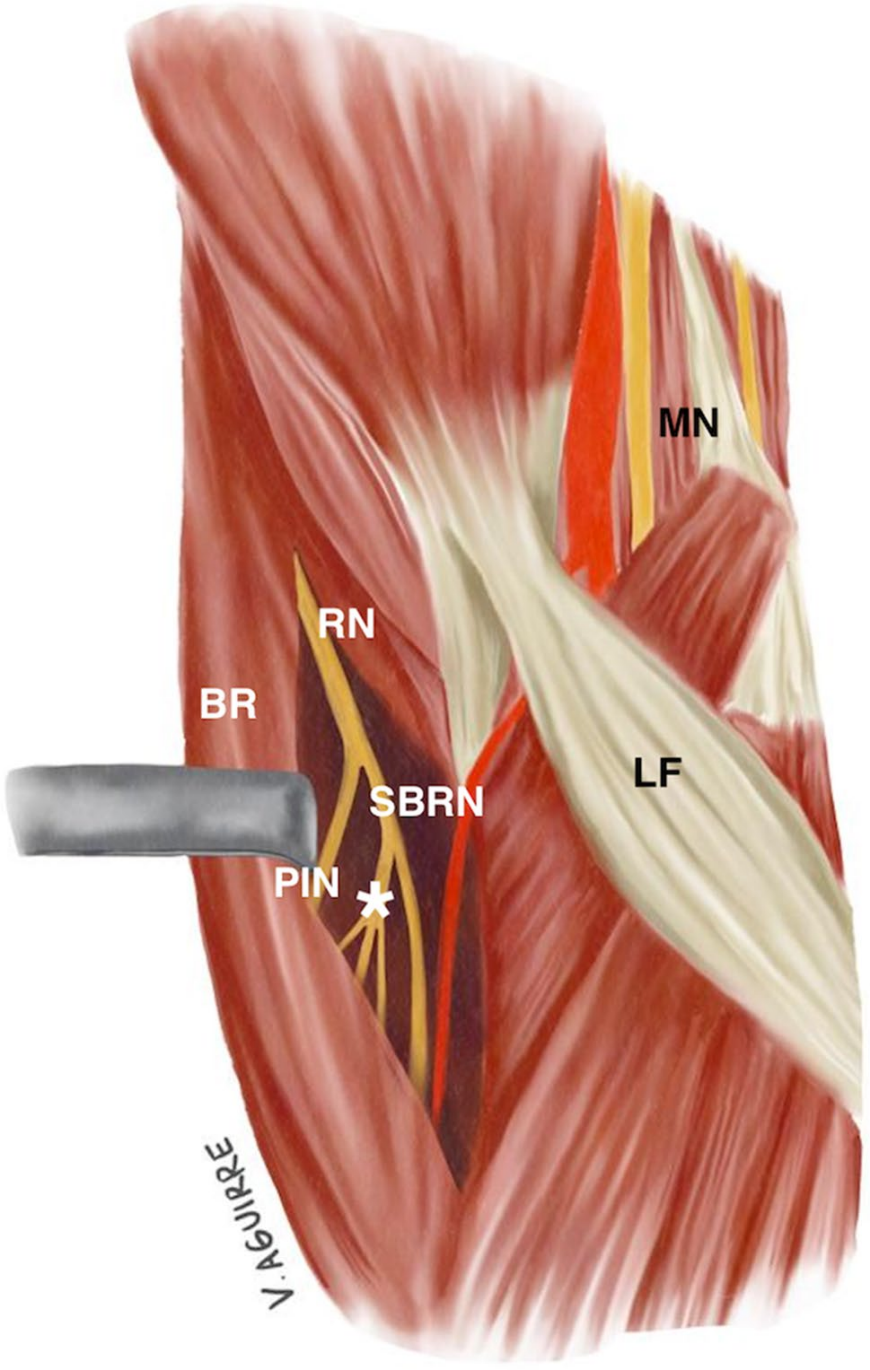
Illustration of the anatomy of the radial nerve and sensory branch of the radial nerve in a right forearm, supinated position, as well as the anatomical relationship to the lacertus fibrosus and median nerve. BR = Brachioradialis; PIN = Posterior interosseous nerve; SBRN = Sensory branch of the radial nerve; * = branches to the extensor carpi radialis brevis (ECRB); LF = lacertus fibrosus; MN = median nerve

In this series, 45% of the cases had both BRS and LS. Multiple concurrent nerve compressions exist and should not be underestimated [13, 14]. When symptoms from more than one site of known nerve compressions are present, each should be correctly assessed and treated. Multiple decompression is a safe procedure with good functional outcomes [13]. The common factor between the BRS and LS is that both are nerve compressions in the proximal forearm, and both compression points are located in vicinity of the distal biceps tendon (Fig. 3). The LS has previously been determined to be a dynamic type of nerve compression [4], and the similar is likely true of BRS. As the majority of our patients (70%) had the nerve compression in the dominant arm, this may further associate BRS with repetitive load/exposure.

Image studies are limited as they usually are inconclusive or normal. Nerve compression can be visualized as an increase in the cross-sectional area or hypoechogenic. For the SBRN, ultrasonographic abnormal images with increased cross-sectional area were described in 50% of the cases [15] (Fig. 2).

Dynamic nerve compression diagnoses are challenging and need to be based on precise physical examination with attention to specific signs and symptoms. As described by Patterson et al. [16], these cases correspond to a Sunderland Zero pathology, where there is no structural damage in the nerve and ischemic nerve block is the main cause of functional loss. Electrodiagnostic studies are of little to no value in these patients as results are usually normal and do not exclude the diagnosis, as abnormal results are only in 8.9% of the cases [17]. This represents an earlier stage in the pathophysiology of nerve compression where the blood-nerve barrier is impaired, resulting in nerve edema, but where no axonal damage or demyelination appears [16]. Diagnosis of dynamic compressions should not be delayed or misdiagnosed, and as electrodiagnostics are generally inconclusive, the clinical examination should routinely include muscle testing to adequately delinate the level of nerve compression.

This study is not without limitations. It is a relatively small case series with a limited follow-up time, where 13 of 20 (65%) completed the postoperative questionnaire. The intraoperative recovery of ECRB strength was, however, observed in all the cases, with statistically significant improvement in functional scores and high satisfaction with surgery.

## Conclusion

In conlusion, proximal forearm compression of the SBRN should be considered in patients with radiovolar forearm pain, loss of wrist extension strength and dysesthesia in the distributions of the SBRN. The surgery is minimally invasive, and executed using walant anesthesia, allows for intraoperative test of strength recovery.

## Supplementary Information

Below is the link to the electronic supplementary material.

Supplementary file1 Clinical examination of proximal forearm compression of the sensory branch of the radial nerve, so called “high Wartenberg” or “brachioradialis” syndrome (MP4 224092 KB)

## Data Availability

The data underlying this article are available in the article and its online supplementary material.
